# The Potential Role of the *Piwi* Gene in the Development and Reproduction of *Plutella xylostella*

**DOI:** 10.3390/ijms241512321

**Published:** 2023-08-01

**Authors:** Dan Liu, Muhammad Asad, Jianying Liao, Jing Chen, Jianwen Li, Xuemei Chu, Senbo Pang, Mubashir Tariq, Anam Noreen Abbas, Guang Yang

**Affiliations:** 1 State Key Laboratory of Ecological Pest Control for Fujian and Taiwan Crops, Institute of Applied Ecology, Fujian Agriculture and Forestry University, Fuzhou 350002, China; 18661591099@163.com (D.L.); axadch@fafu.edu.cn (M.A.); 2818368960@fafu.edu.cn (J.L.); 1200203002@fafu.edu.cn (J.C.); li1928999798@163.com (J.L.); 15165546235@163.com (X.C.); pangsenbo@163.com (S.P.); mubashirtariq77@gmail.com (M.T.); anam.noreen99@yahoo.com (A.N.A.); 2 Joint International Research Laboratory of Ecological Pest Control, Ministry of Education, Fuzhou 350002, China; 3 Ministerial and Provincial Joint Innovation Centre for Safety Production of Cross-Strait Crops, Fujian Agriculture and Forestry University, Fuzhou 350002, China; 4 Key Laboratory of Integrated Pest Management for Fujian-Taiwan Crops, Ministry of Agriculture, Fuzhou 350002, China; 5 Key Laboratory of Green Pest Control, Fujian Province University, Fuzhou 350002, China

**Keywords:** PIWI protein, oviposition, hatchability, CRISPR/Cas9, RNAi, diamondback moth

## Abstract

Piwi proteins play a significant role in germ cell development and the silencing of transposons in animals by associating with small non-coding RNAs known as Piwi-interacting RNAs (piRNAs). While the *Piwi* gene has been well characterized in various insect species, the role of the *Piwi* (*PxPiwi*) gene in the diamondback moth (*Plutella xylostella*), a globally distributed pest of cruciferous crops, remains unclear. Expression analysis demonstrated the upregulation of *PxPiwi* in pupae and testes. Furthermore, we generated a *PxPiwi*-knockout mutant using CRISPR/Cas9 technology, which resulted in a significantly prolonged pupal stage and the failure of pupae to develop into adults. Additionally, the knockdown of *PxPiwi*, through RNA interference (RNAi), led to a substantial decrease in the oviposition and hatchability of *P. xylostella*. These findings indicate that *PxPiwi* is specifically expressed and essential for the development and reproduction of *P. xylostella*. This is the first report indicating the involvement of the *Piwi* gene in the development of lepidopteran insects, except for reproduction and germ cell development, which provides a foundation for future investigations into the functions of *PxPiwi*.

## 1. Introduction

PIWI-interacting RNAs (piRNAs) are essential for germ cell development and the silencing of transposon elements [1]. piRNAs are small RNAs that interact with PIWI proteins and have been well-identified in several species [2,3,4]. piRNAs bind to PIWI proteins to form complexes that act on DNA, thereby affecting DNA histone methylation and playing a critical role in the developmental processes associated with sexual reproduction [5].

The piRNA pathway is classified based on Argonaute protein interactions and biogenesis [6]. The Argonaute protein family is a superfamily that has been further classified into two subfamilies, Argonaute (Ago) and PIWI. PIWI proteins are composed of an N-terminal PAZ (PIWI-Argonaute-Zwille) and MID (middle) and PIWI domains [7], among which, the PAZ and PIWI domains are the most dominant and conserved [8,9]. The PAZ domain provides binding sites for small RNAs and regulates gene silencing at the post-transcription level [10]. The PIWI domain has a similar structure to ribonucleases H (RNaseH) and is the center of enzymatic cleavage activity in the RNA-mediated silencing complex [11,12]. Moreover, the PIWI domain contains a slicer-active site, which cleaves target RNA complementary to small RNA during the silencing process [13,14]. In most species, the expression of PIWI proteins is restricted to the reproductive tissues (ovaries and testes) and is highly conserved [4]. Previous studies have confirmed that PIWI proteins have similar roles in different species, such as the regulation of gametogenesis, the maintenance of germline stem cells, and the regulation of transposon silencing. The regulation of gametogenesis is the most important function of PIWI proteins, and the knockout of *Piwi* causes severe impairment to the fertility of *Caenorhabditis elegans* [15], flies [16,17], and mammals [18]. The knockdown of *PmPiwi1* and *PmPiwi2* causes a significant reduction in sperm count in shrimp [19]. The *Piwi* genes *prg-1* and *prg-2* also play crucial functions in maintaining germ cells in *C. elegans* [15]; in zebrafish, the knockout of two *Piwi* genes, *Ziwi* and *Zili*, exhibits germ cell failure, and adult mutant germ cells show an abnormal apoptotic phenotype [2,20]. The deletion of the *Piwi* gene in *Drosophila melanogaster* leads to the accumulation of retrotransposons in the spermatozoa and ovaries, and mutations in *Piwi* lead to an increase in retrotransposon transcripts, indicating a vital role in germline transposon silencing in *D. melanogaster* [21], which is similar in mice [22].

The members of the PIWI subfamily vary between the insect species. For example, three members of PIWI in *D. melanogaster* and two in *Bombyx mori* have been identified and characterized [1,17,23]. To date, only one gene, *Ago3*, has been identified and characterized in *P. xylostella* [24], which is a notorious pest of cruciferous crops that annually causes approximately USD 5 billion in damage and control [25,26]. Therefore, new approaches to pest governance are urgently needed, and germline-specific genes and their promoters may provide a way to develop an effective genetic control strategy for *P. xylostella*. In this study, we discovered the *Piwi* gene in *P. xylostella* and performed comprehensive functional characterization using different approaches to evaluate its role in reproduction and development.

## 2. Results

### 2.1. Identification of Piwi Gene in P. xylostella and Phylogenetic Analysis

**Figure 1:**
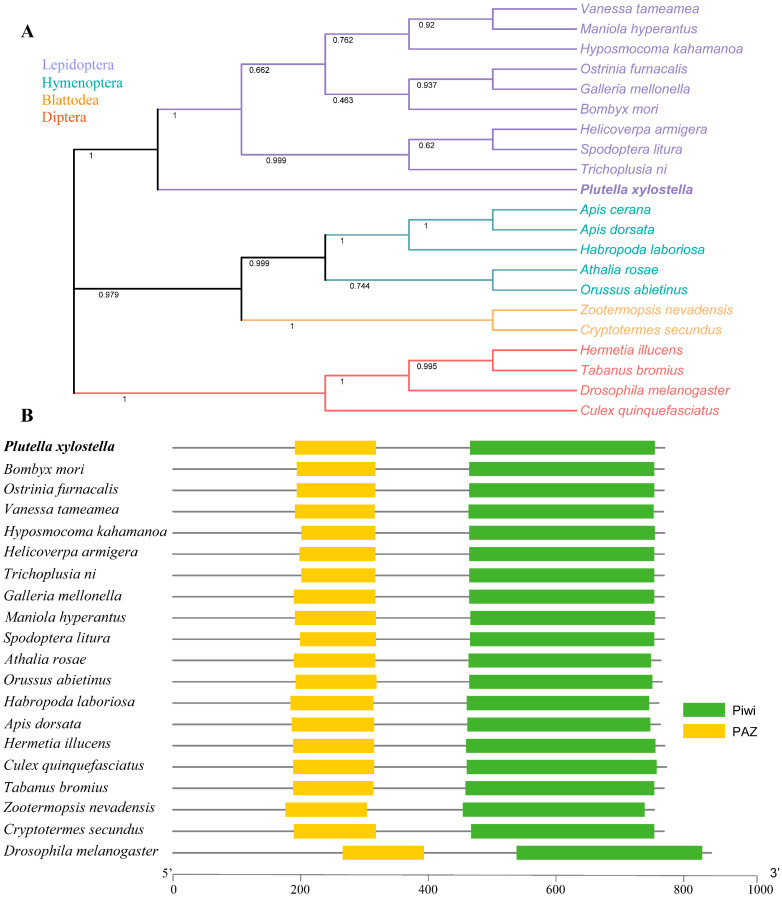
Phylogenetic relationship and domain structure of Piwi. (**A**) Phylogenetic tree of Piwis of 21 different insect species from four orders. Insects from the four orders were emphasized using different colors. (**B**) Schematic representation of conserved domains of Piwi proteins.

First, we obtained the *D. melanogaster* Piwi protein sequence (AGL81534.1) from the NCBI and blasted it against the *P. xylostella* genome. A highly similar gene sequence (Px000788) was obtained, which is considered the *Piwi* gene of *P. xylostella* (Table S2). *PxPiwi* contained 2160 bp open-reading-frame, encoding 779 amino acids. The molecular weight and theoretical isoelectric point of PxPiwi were determined to be 88.08 Kda and 9.01, respectively. PxPiwi protein contained two conserved domains of the PAZ domain (191–318 aa) and the PIWI domain (465–757 aa) (Figure 1 and Figure S1). Additionally, five glycosylation sites (N-X-S-T) and 122 putative phosphorylation residues were identified in the PxPiwi protein (Figure S1). To determine the evolutionary relationship, a phylogenetic tree was constructed, comparing the amino acid sequence of PxPiwi with 21 different insect species belonging to four different orders. The phylogenetic analysis revealed that PxPiwi was highly conserved in different species, and PxPiwi was clustered into the same clade with Piwis of other lepidopteran insects (Figure 1). Furthermore, the multiple alignments of amino acid sequences of five lepidopteran species revealed that Piwi shared a similarity of about 87% within those insect species (Figure S2).

### 2.2. Stage- and Tissue-Specific Expression Profile of Piwi Gene in P. xylostella

**Figure 2:**
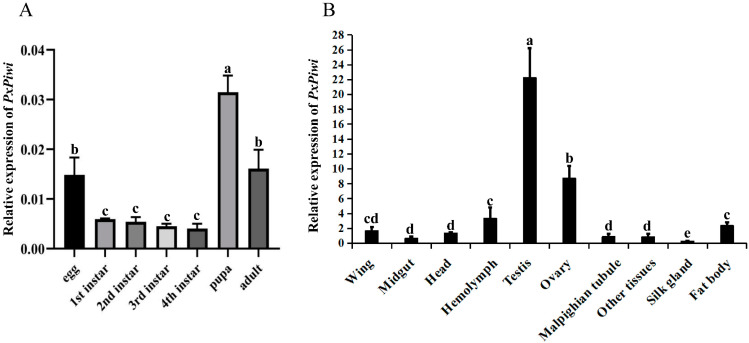
The expression profile of the *PxPiwi* gene at different stages and in different tissues. (**A**) The relative expressions of the *PxPiwi* gene at different stages; (**B**) the relative expressions of the *PxPiwi* gene in different tissues obtained from the pupal stage. The bars indicate the mean ± SEM of five biological replications. Means sharing the same letters indicate no significant difference, with values of *p* < 0.05 from each other.

To determine the relative expressions of *PxPiwi* in various developmental stages and tissues, RT-qPCR was performed. The findings revealed that *PxPiwi* exhibited significantly high relative expression levels in pupae, followed by adults and embryos. However, the expression of *PxPiwi* was notably lower in larvae compared with embryos, pupae, and adults (Figure 2A). To gain further insight into its expression patterns, we investigated the relative expressions of *PxPiwi* in various tissues of pupae. The results demonstrated significantly higher expression levels in reproductive tissues, particularly in the testes, followed by the ovaries, compared with other tissues such as the midgut, head, fat body, and Malpighian tube (Figure 2B).

### 2.3. CRISPR/Cas9-Mediated Mutagenesis of PxPiwi

**Figure 3:**
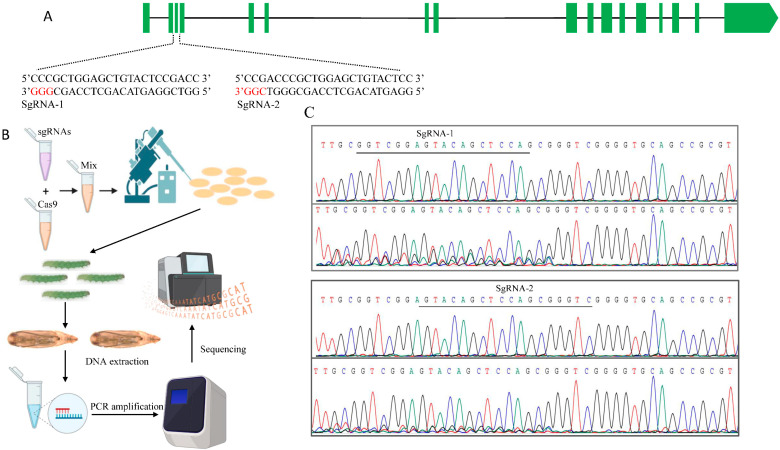
CRISPR/Cas9-mediated mutagenesis of *PxPiwi* gene. (**A**) The gene structure (intron and exon distribution) of the *PxPiwi* gene and two sgRNA sequences targeting the exon 2 of the *PxPiwi* gene. The red color base pairs represent the protospacer-adjacent motif (PAM) sequences of sgRNAs. (**B**) The schematic flow of embryo microinjection and mutation detection through sequencing. (**C**) Sequence chromatogram for detection of mutation at the target site.

The *PxPiwi* gene consisted of 15 exons and 14 introns with a length of 16,654 bp. Two sgRNAs were designed targeting exon 2 of *PxPiwi* (Figure 3). A total of 212 freshly laid embryos were injected with a mixture containing sgRNA1, sgRNA2, and Cas9 proteins, within 30 min of oviposition (Figure 3).

**Figure 4:**
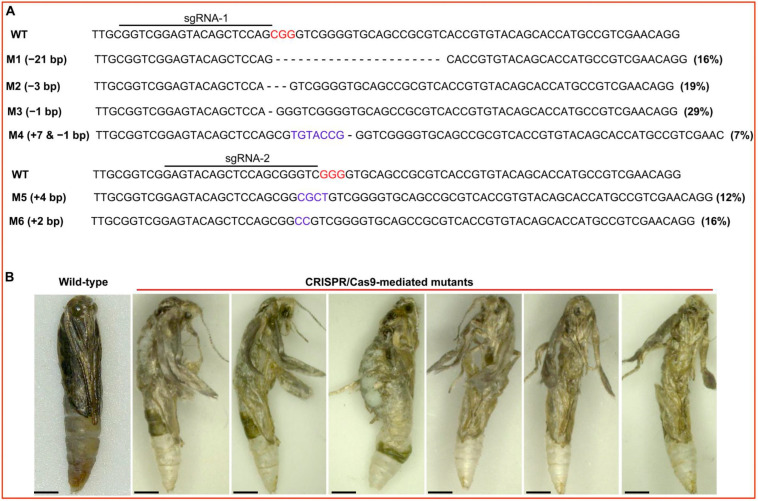
Indel mutation at *PxPiwi* target site and abnormal pupal phenotypes caused by knockout of *PxPiwi* gene using CRISPR/Cas9 technique. (**A**) Six types of indel mutations. The red base pairs represent the PAM sequence of the sgRNA target site; dashes (−) highlight the deletion of nucleotides; and blue-colored base pairs indicate the insertion of nucleotides at the target site. (**B**) The abnormal phenotypes during pupa to adult molting. All pupae pictures were taken with a stereoscope by using a 500 μm scale bar.

Out of these injected embryos, a total of 98 individuals survived up to the pupal stage, with a survival rate of 46.22%. A total of 44 pupae became adults, and the remaining 54 pupae exhibited abnormal death during the molting process from pupa to adult. The mutations of all surviving pupae and adults were confirmed through PCR and sequencing (Figure 4A). All 54 abnormal pupae were mutated at both sgRNA target sites, with a total mutation rate of 55.1% (54 out 98) (Figure 4B). Six types of indel (deletion or insertion) mutations were observed, including deletions of −21 bp, −3 bp, and −1 bp and insertions of +7 bp, +4 bp, and +2 bp (Figure 4A). Furthermore, the abnormal pupae that failed to become adults exhibited similar abnormal phenotypes, such as abnormal leg and wing development, deformed abdomens, and abnormal head development (Figure 4B). Interestingly, two homozygous editing types of −3 bp and +7 −1 bp appeared in the abnormal pupae (Figure S3). Therefore, these findings strongly suggest that *PxPiwi* is essential for the survival and molting of pupae.

### 2.4. Effect of PxPiwi Mutation on the Developmental Duration of Plutella xylostella

**Figure 5:**
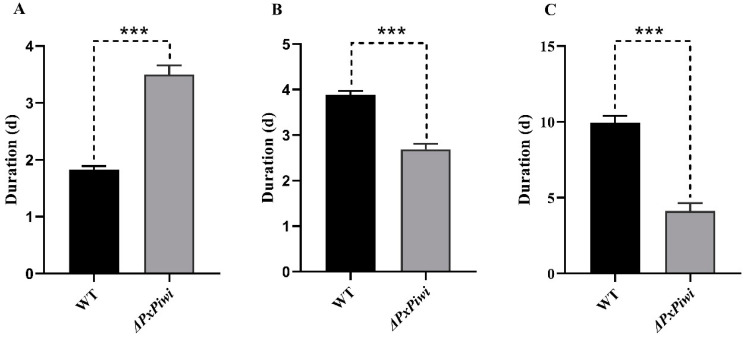
Comparison of developmental duration between *PxPiwi* mutants (Δ*PxPiwi*) and wild-type individuals (WT). (**A**) The 4th-instar larval duration; (**B**) the pupal duration; (**C**) the adult duration. Bars highlight the mean ± SEM of more than five replications. A significant difference at *p* < 0.001 is denoted by ***.

For the *PxPiwi* mutant, the developmental time from hatching to the fourth-instar larvae was normal. In *PxPiwi* mutants, the duration of the fourth-instar larval stage was noticeably prolonged compared with wild-type individuals (Figure 5A), while the duration of both the pupal and adult stages in the *PxPiwi* mutants was significantly shorter than that observed in the wild type (Figure 5B,C). These findings suggest that *PxPiwi* plays a significant role in the development of *P. xylostella*.

### 2.5. Effect of RNAi-Mediated Knockdown of PxPiwi on Reproduction of P. xylostella

**Figure 6:**
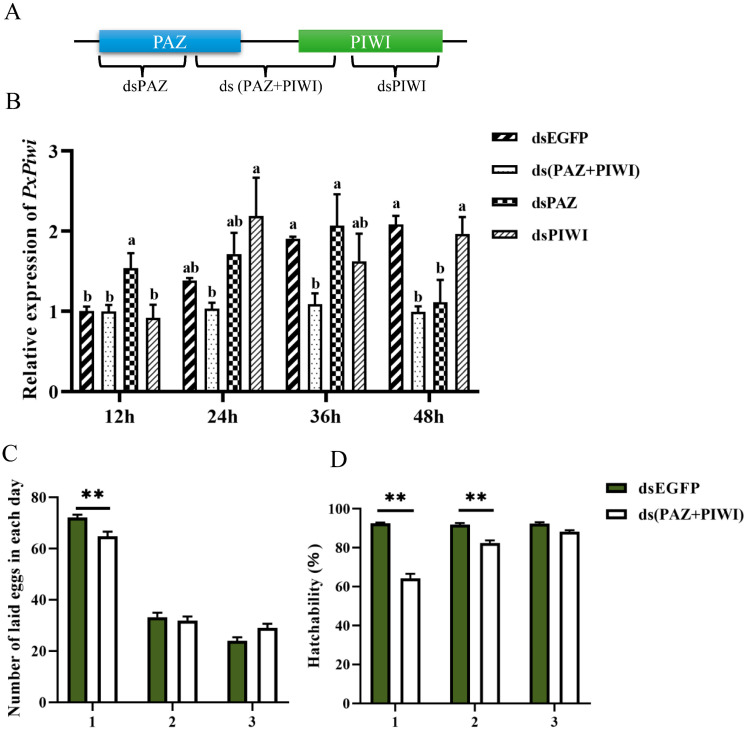
RNAi of *PxPiwi* and its effects on oviposition and hatchability of *P. xylostella*. (**A**) Schematic representation of three dsRNAs targeting the two conserved regions of *PxPiwi*; (**B**) relative expression levels of *PxPiwi* at different time points after the injection of different dsRNAs at the pupal stage; (**C**) the number of eggs laid by the female after the injection of ds(PAZ+PIWI) on each day; (**D**) hatchability of eggs laid by the females after the injection of ds(PAZ+PIWI) on each day. ds(PAZ+PIWI): the dsRNA targeting the PAZ and PIWI domains of *PxPiwi*; ds*EGFP*: the dsRNA targeting *EGFP*. The bars highlight mean ± SEM; the means sharing the same lowercase letters are not significantly different from each other at *p* < 0.05, and ** represents the significant difference at *p* < 0.01.

In order to confirm the involvement of the *PxPiwi* gene in the reproduction of *P. xylostella*, RNA interference (RNAi) was carried out. Three distinct double-stranded RNAs (dsRNAs) were designed and synthesized to target specific domains: dsPAZ for the PAZ domain, dsPIWI for the PIWI domain, and ds(PAZ+PIWI) for both domains (Figure 6A). dsEGFP was used as a control. All these synthesized dsRNAs were individually injected into the pupae of *P. xylostella*, and injected pupae were further used to determine the silencing efficiency of three dsRNAs at different time points post-injection. The findings indicated that there was no notable difference in the expression of the *PxPiwi* gene between the ds(PAZ+PIWI) group, the dsPIWI group, and the control group at 12 hours post-injection; meanwhile, the expressions of *PxPiwi* were significantly upregulated in the dsPAZ group (Figure 6B). The expressions of *PxPiwi* showed no significant difference between the dsPAZ, dsPIWI, and control groups at 24 h or 36 h post-injection. However, at 36 h post-injection, the ds(PAZ+PIWI) group exhibited a significant downregulation in the relative expression of *PxPiwi* compared with the control group (Figure 6B). Likewise, at 48 h post-injection, the relative expression level of *PxPiwi* was remarkably decreased in both the ds(PAZ+PIWI) and dsPIWI groups, while no significant change was observed in the dsPAZ group when compared with the control group (Figure 6B). These results indicated the ds(PAZ+PIWI) group showed high RNAi efficiency compared with the other groups (Figure 6B). Thus, the ds(PAZ+PIWI) treatment group was further selected to evaluate the effects of the knockdown of *PxPiwi* on the fecundity and reproduction of *P. xylostella*. 

To determine the effects of *Pxpiwi* knockdown on fecundity and the hatching rate, the adults that emerged from dsRNA-injected pupae were separately crossed with WT adults of the opposite sex. The findings showed that the eggs laid by female *P. xylostella* injected with ds(PAZ+PIWI) were significantly lower than those injected with dsEGFP on the first day after mating (Figure 6B). No significant changes were observed in the number of eggs laid on the second or third days after mating (Figure 6B). Therefore, these results indicate that *Piwi* suppression significantly reduced the fecundity of *P. xylostella*.

Subsequently, we examined the hatching rate of eggs laid by the female after the injections of ds(PAZ+PIWI). The results revealed a significant decrease in the hatching rate of eggs laid by treated females on the first and second days compared with eggs from the control group; meanwhile, on the third day, no significant difference in the hatching rate was observed between the injected and control groups (Figure 6C). Thus, the RNAi-mediated knockdown of *PxPiwi* significantly reduced the hatchability of *P. xylostella.*

## 3. Discussion

In the present study, we identified and cloned the *PxPiwi* gene based on a *P. xylostella* genomic database and deduced amino acids showed that *PxPiwi* contains two conserved domains, PAZ and PIWI. The PAZ domain is considered a specific binding component that provides binding sites for small RNAs generated from Rnase III-like enzymes such as Dicer [27]. The presence of the PIWI domain is crucial for the RNAi-splicing processes [28]. Extensive studies have shown that both the PAZ and PIWI domains are essential for the proper functioning of Piwi-related genes in transpose silencing and germ cell development [29,30,31].

The relative expressions of *PxPiwi* were limited to germ tissues, as it has been demonstrated in other species that Piwi subfamily genes are profoundly expressed in germ tissues [32]. *PxPiwi* was highly expressed in the testes and ovaries of pupae. These findings align with prior investigations conducted on *D. melanogaster* and *B. mori*, where the expression of the *DmPiwi* and *Bmsiwi* genes was found to be highly expressed in tissues associated with germ cells [1,33], suggesting that PxPiwi may have some role in germ cell development and reproduction in *P. xylostella*. Moreover, previous studies have reported the involvement of Piwi subfamily genes in the development of germ cells in zebrafish and mice [32,34]. The *PxPiwi* expression pattern was remarkedly varied between the ovaries and testes and highly expressed in the testes, indicating that *PxPiwi* may have some roles in testis development and spermatogenesis, as it has been reported that *Bmsiwi* is a crucial gene in the spermatogenesis of *B. mori* [1].

The knockout of *PxPiwi* affected pupal development, the abnormal eclosion from pupa to adult, and the abnormal adult phenotype. Interestingly, we were not expecting that the knockout of *PxPiwi* would have any effects on development and pupal eclosion since extensive studies have shown that Piwi subfamily genes have a role in germ cell development and spermatogenesis in lepidopteran insects [35]. Despite the known germline-restricted functions of Piwi, the role of these genes in somatic tissue development has been explored in model insects such as *D. melanogaster*. PIWI proteins have a somatic role during the early embryogenesis of *D. melanogaster*, to which *Piwi* mutant female embryos are somatically lethal [17,31,36]. Moreover, the knockout of the *AUB* gene (the Piwi subfamily gene) leads to abdominal deletion in females and no germ cell formation [37]. Piwi proteins exhibit epigenetic effects in somatic tissues such as the eyes and salivary glands by binding to available chromosomes in these tissues in the larvae and adults of *D. melanogaster*. Furthermore, these genes play a role in the development of somatic tissues in *D. melanogaster* [30,38,39,40]. A CRISPR/Cas9-mediated knockout of *BmSiwi* and *BmAgo* was performed on the BmN cell lines of *B. mori,* and it was found that both proteins are essential for the cell survival and development of the BmN cell line [41]. Although *PxPiwi* expresses in germ-related tissues, it also plays a role in somatic tissue formation and is necessary for the survival of *P. xylostella*. Since the *PxPiwi*-knockout mutant did not survive, we could not conduct further experiments to evaluate the role of *PxPiwi* at this stage. Therefore, further studies can be planned to develop knockout strains through the CRISPR/Cas9 binary system [42], which could help find out the actual role of the *PxPiwi* gene in *P. xylostella*.

Next, the RNAi-mediated knockdown of the *PxPiwi* gene was performed to determine if it has a role in the reproduction of *P. xylostella*. Two conserved regions (PAZ and PIWI) were combined and separately targeted, and *PxPiwi* expressions were only downregulated in individuals with the combined target, which indicated that both PAZ and the PIWI conserved region are important for *Piwi* gene regulations. As previously described in different species, both the PIWI and PAZ domains are important for the expression and regulation of *Piwi*-related genes [29,43]. The knockdown of the *PxPiwi* gene significantly decreased the egg production and hatchability of *P. xylostella*. *Piwi*-related genes have been widely studied; they are germline-specific and play a vital role in gonadal tissue formation. For example, in zebrafish, mice, and *B. mori*, *Piwi* genes are responsible for spermatogenesis [1,32,34], which ultimately has effects on reproduction. These findings highlight that *PxPiwi* is also essential for the reproduction of *P. xylostella*.

## 4. Materials and Methods

### 4.1. Insect Rearing

The Genova88 strain (artificial diet) was utilized in this study. The process of diet preparation and rearing methodology has been extensively documented in previous literature [44,45]. Larvae were raised in a controlled environment within the Institute of Applied Ecology at Fujian Agriculture and Forestry University, maintaining specific conditions, including a temperature of 26 ± 1 °C, relative humidity of 65%, and a photoperiod of L:D = 16:8. Following pupation, the pupae were collected and transferred to a mating box, where cotton soaked in a 10% honey solution and an egg card coated with rapeseed powder were provided.

### 4.2. Total RNA Extraction, cDNA Preparation, and Cloning of Piwi Gene

The adults of *P. xylostella* were used to isolate total RNA using the RNA Simple Total RNA Kit (TIANGEN Biotech, Beijing, China) as per the provided protocol. The isolated RNA was then subjected to the DNAase I treatment (TIANGEN Biotech, Beijing, China) to eliminate DNA contaminants. Subsequently, first-strand cDNA was synthesized using the extracted RNA as a template with the one-step RT-PCR kit (Takara Biomedical Technology, Beijing, China) following the provided protocol. The full-length open reading frame (ORF) of the *PxPiwi* gene was obtained from the *P. xylostella* genomic database [46]. A pair of primers were designed (Table S1) to amplify the ORF of *PxPiwi*, and the prepared cDNA was employed as the template in a PCR reaction mixture. The Super-Fidelity DNA Polymerase kit (Vazyme, Nanjing, China) was used to prepare the PCR reaction mixture. The amplified PCR product was subsequently purified, ligated into the PJET1.2 blunt-end vector (Thermo Scientific, Waltham, MA, USA) following the standard protocol, and confirmed through sequencing.

### 4.3. Phylogenetic Analysis

For the purpose of investigating the evolutionary relationship, the amino acid sequences of 21 distinct insect species from four distinct orders were acquired from the NCBI (Table S3). These sequences were then aligned with the amino acid sequence of PxPiwi using the CLUSTALW program. The aligned sequences were then utilized to generate a phylogenetic tree employing the maximum likelihood method with a bootstrap value of 1000 using the MEGA 10 software. The resulting tree image was further analyzed and adjusted using the online tool iTOL (http://itol.embl.de/ accessed on 28 February 2023).

### 4.4. Expression Analysis

Total RNA was isolated from different stages of eggs, first-instar larvae, second-instar larvae, third-instar larvae, fourth-instar larvae, pupae, and adults and from different tissues of the wings, midgut, head, hemolymph, ovaries, testes, Malpighian tubule, fat body, and silk glands, as well as other tissues. cDNA was prepared by following the abovementioned protocol. The qPCR reaction mixtures were prepared using gene-specific primers and the GoTaq^®^ qPCR master mix (Promega, Madison, WI, USA) (Table S1). The endogenous reference gene, *ribosomal protein L32* (*PxRPL32*; GenBank acc. No. AB180441) was used to normalize the expression of the target gene. Three biological replications and three technical replications were performed. The relative gene expressions were calculated by using the 2^ΔCt^ method.

### 4.5. Design and In Vitro Synthesis of sgRNA

sgRNA sequences were designed following the principle of 5′-GG-(N)18-NGG-3′ [44] using the online sgRNA design tool CHOPCHOP (https://chopchop.cbu.uib.no/; accessed on 3 March 2023), and CRISPR RGEN tools (https://www.rgenome.net/cas-offinder/; accessed on 3 March 2023) were used for mismatches. Finally, two sgRNA targets were successfully designed on exon 3 of the *P. xylostella Piwi* gene. Oligonucleotides containing the T7 promoter and sgRNA sequences were designed (Table S1). To generate an in vitro template for sgRNA transcription, PCR amplification was performed using the Super-Fidelity DNA Polymerase (Vazyme, China) and the designed oligonucleotides (Table S1). The resulting PCR products were visualized via agarose gel electrophoresis, purified using the Gel Extraction Kit (Omega, Norcross, GA, USA), and then utilized as the template for the in vitro transcription of sgRNAs. Finally, the sgRNAs were transcribed in vitro using the purified PCR products as the template with the HiScribeTM T7 Quick High Yield RNA Synthesis Kit (New England Biolabs, Ipswich, MA, USA) and following the recommended protocol.

### 4.6. Germline Microinjection

For germline microinjections, a 10 μL mixture was prepared containing a final concentration of 300 ng/μL of GenCrisprCas9-N-NLSNulease and 150 ng/μL of each of the two sgRNAs (total 300 ng/μL of sgRNAs concentration) [47]. Further, the prepared mixture was incubated for 20 min at 37 °C and directly used for injections. The eggs were collected using parafilm paper within 20 min of oviposition. Within 30 min of oviposition, the collected eggs were injected using a microinjection setup comprising an IM 300 microinjector (Narishige, Tokyo, Japan) and an SZX16 stereoscopic microscope (Olympus, Tokyo, Japan). The injected eggs were then placed in a Petri dish for hatching. After hatching, the larvae were fed an artificial diet.

### 4.7. Phenotype Screening and Mutation Detection

The phenotypes of all surviving pupae of the G_0_ generation were observed by using a stereoscope equipped with a white light filter and a camera. For mutation detection, genomic DNA was extracted from all G_0_ individuals by using the Blood/Cell/Tissue Genomic DNA Extraction Kit (Tiangen, China). A specific primer was designed near the sgRNA target to screen the mutants (Table S1). PCR reactions were conducted using genomic DNA as the template for amplification, along with specific primers and GoTaq^®^ Green Master Mix (Promega, USA). The resulting PCR products were subsequently subjected to sequencing without any additional steps. Overlapping peaks or base-deletion single peaks near the PAM junction of the target in the sequencing map were considered mutations. Furthermore, to detect mutation types, the amplified PCR products from mutants were further cloned into blunt-end vector and subjected to sequencing.

For the evaluation of development duration, more than five *PxPiwi* mutants and WT individuals were selected. All stages from hatching to adult were closely observed, and the duration of each stage was noted. The duration of each stage of the *PxPiwi* mutants was compared with the WT individuals, and we calculated the differences in developmental duration between the WT strain and the *PxPiwi* mutants.

### 4.8. Design and In Vitro Synthesis of dsRNAs

Based on the verified *PxPiwi* sequence, the primers for the PIWI domain; the PAZ domain; the overlap region of the PIWI domain and PAZ domain; and the EGFP gene were designed using Primer Premier 5.0. To prepare the template for dsRNA synthesis, the 5′ end of each designed primer was modified to include the T7 promoter sequence (Table S1). The in vitro transcription template for dsRNA was generated through PCR amplification using the Super-Fidelity DNA Polymerase (Vazyme, China). The resulting PCR products were purified using the Gel Extraction Kit (Omega, USA). Finally, the T7 RiboMAXTM Express RNAi System (Promega, USA) was utilized for the in vitro synthesis of the dsRNAs.

### 4.9. Microinjections of dsRNAs

The feather coats of the pupae were removed with tweezers. The pupae were injected with different dsRNAs of the target gene *PxPiwi* and the dsRNA of EGFP as a control by using the Nanoject III Programmable Nanoliter Injector (Drummond, Birmingham, Alabama USA), and the injection point was kept at the tail of each pupa. A total of 600 ng of dsRNA was injected into each pupa. The injected pupae were placed in a centrifuge tube with holes.

### 4.10. Sample Collections and Expression Analysis after Microinjections

Samples were collected at four different time points, 12, 24, 36, and 48 h, after injection, with five biological replicates for each time point. RNA was extracted from the collected samples and utilized to generate first-strand cDNA following the previously mentioned protocol. To analyze the expression of *PxPiwi* after dsRNA treatment, qPCR was conducted using gene-specific primers and GoTaq^®^ qPCR Master Mix. The expression levels were normalized using the housekeeping gene *PxRPL32*, and the relative expression was calculated using the 2^ΔΔCt^ method. The remaining injected individuals were used to mate with opposite wild-type individuals to determine the oviposition and hatchability.

### 4.11. Bioassay for Oviposition and Hatchability

After the eclosion of dsRNA-injected pupae, the adults were separately crossed with the newly emerged wild-type adults of the opposite sex in a mating cup containing an egg card. The control group consisted of pupae that were injected with dsEGFP. The egg cards were collected and replaced at the same time every day. The daily fecundity and egg hatching within 3 d after mating were recorded, and the daily hatching rate was calculated. During this period, the adults were fed with honey.

### 4.12. Statistical Analysis

All experimental data were stored in Microsoft Excel. Statistical analysis was conducted using the SPSS 25.0 software, employing one-way ANOVA followed by Tukey’s test to compare the expressions of *PxPiwi* at different stages and tissues, RNAi efficiency, oviposition, hatching rate, and developmental duration, from 4th-instar larvae to adults. Graphical illustrations were created using GraphPad Prism 8.

## 5. Conclusions

In conclusion, our study highlights the significance of the *PxPiwi* gene, particularly concerning the development and reproduction of *P. xylostella*. This is the first report indicating the involvement of the *Piwi* gene in the development of a lepidopteran insect, in addition to its role in reproduction. Moreover, these findings lay a foundation for future investigations on the gene functions of *PxPiwi*.

## Data Availability

All data are available in the manuscript and the Supplementary File.

## Supplementary Materials

The supporting information can be downloaded at https://www.mdpi.com/article/10.3390/ijms241512321/s1.
