# Supplementary material for: The Potential Role of the Piwi Gene in the Development and Reproduction of Plutella xylostella
Source: Int J Mol Sci. 2023 Aug 1;24(15):12321. doi: 10.3390/ijms241512321 (PMC10418840; doi:10.3390/ijms241512321)
Supplement: Supplementary file 1 [file ijms-24-12321-s001.zip › ijms-2491099-supplementary.pdf]

Supplementary Information

Table S1 Primers used for qRT-PCR

Primer name	Primer sequence
Pxpiwi-qPCR-F	ACAAGGTGCTGCGAATGGAT
Pxpiwi-qPCR-R	ACGCGGTACGTCTTCTTGTT
RPL32-qPCR-F	CAATCAGGCCAATTTACCGC
RPL32-qPCR-R	CTGGGTTTACGCCAGTTACG
dsPIWI-F	TAATACGACTCACTATAGGGGCTGGACCAGGGAGATGAGA
dsPIWI-R	TAATACGACTCACTATAGGGTTGCTGGTCATGTTGCGCGC
dsPAZ-F	TAATACGACTCACTATAGGGCGAGTACTCGCAAAGCAAGG
dsPAZ-R	TAATACGACTCACTATAGGGTTGGTGCGCATCTCGTCCGA
ds(PIWI+PAZ)-F	TAATACGACTCACTATAGGGCCAGATCAGGATCCACGACG
ds(PIWI+PAZ)-R	TAATACGACTCACTATAGGGGGCGACCCTTGATCTTCACC
dsEGFP-F	TAATACGACTCACTATAGGGAGGAGCGCACCATCTTCTTC
dsEGFP-R	TAATACGACTCACTATAGGGGACTGGGTGCTCAGGTAGTG
Pxpiwi-qPCR-F	ACAAGGTGCTGCGAATGGAT
Pxpiwi-qPCR-R	ACGCGGTACGTCTTCTTGTT
RPL32-qPCR-F	CAATCAGGCCAATTTACCGC
RPL32-qPCR-R	CTGGGTTTACGCCAGTTACG
Pxpiwi-sgRNA1-F	TAATACGACTCACTATAGGTCGGAGTACAGCTCCAGCGTTTTAGAGCTAGAA ATAGCAAGTTAAAATAAGGCTAGTCC
Pxpiwi-sgRNA2-F	TAATACGACTCACTATAGGAGTACAGCTCCAGCGGGTGTTTTAGAGCTAGAA ATAGCAAGTTAAAATAAGGCTAGTCC
sgRNA-R	AAAAGCACCGACTCGGTGCCACTTTTTCAAGTTGATAACGGACTAGCCTTAT TTTAACTTGCTATTTCTAGCTCTAAAA
The underlined is T7 promoter sequence for in vitro transcription; the bold bases are enzyme cutting sites and red color bases represent the sgRNA sequence.	

Table S2 Blast results for identification of *piwi* gene in *Plutella xylostella*

No.	Gene ID	Coverage	Identity	E-value	Score	Annotation
1	Px000788	97.29%	42.65%	0.0	657	Protein piwi
2	Px011435	93.28%	30.27%	e-125	398	Aubergine (Piwi-like protein)
3	Px011436	85.21%	27.19%	2e-63	224	Piwi-like protein 2 (only contain PAZ domain)
4	Px012889	86.21%	23.67%	3e-25	109	Protein argonaute-2
5	Px006072	31.48%	28.57%	2e-24	109	Protein argonaute-4

Table S3 Detail information of *piwi* genes in different species.

Order	Species Name	Gene Id
Lepidoptera	*Plutella xylostella*	XP_037975009.1
	Bombyx mori	NP_001098066.2
	Ostrinia furnacalis	XP_028164126.1
	Vanessa tameamea	XP_026500179.1
	Hyposmocoma kahamanoa	XP_026315894.1
	Helicoverpa armigera	XP_021190043.1
	Trichoplusia ni	XP_026732466.1
	Galleria mellonella	XP_026764636.1
	Maniola hyperantus	XP_034827325.1
	Spodoptera litura	XP_022818139.1
Hymenoptera	*Athalia rosae*	XP_012250886.1
	Orussus abietinus	XP_023290765.1
	Habropoda laboriosa	KOC68594.1
	Apis cerana	XP_016916700.1
	Apis dorsata	XP_006616743.1
Blattodae	*Hermetia illucens*	XP_037908712.1
	Tabanus bromius	ALC79956.1
Diptera	*Culex quinquefasciatus*	EDS35693.1
	Culex quinquefasciatus	EDS35736.1
	Tabanus bromius	ALC79957.1
	Zootermopsis nevadensis	KDR24113.1
	Cryptotermes secundus	XP_023703405.1
	Drosophila melanogaster	AGL81534.1

TTILRTKPANVTTKQGTYGTPLELFANYFTVETTPQWRLYQYHVDFQPEEDRTMVRKSLLRVHKNTLGGYLFDGMVLYTV 80

TRLHPDPLELYSDRKEDGERMRMLIKLTCDVAPGDYHYIQVFNIIIRKCFHALNLQLVGRDFFDAEAKVDIPEYKLQVWP 160

GYKTTINQYEDRLLMVAEIAHKVLRMDTILQMLNEYSQSKGSQYKRMFLEDVVGKIVMTDYNKKTYRVDDVKWDESPQCT 240

FVMRGEAISYVDYYFKKYQIRIHDVKQPLLVSRSKPRDIRAGMPELVYLVPELCRQTGLSDEMRTNFQLMKALDVHTKLG 320

PDARIQKLMSFNRRLTQTPEVVQELKDWEMKLSTTLVKIKGRHMPPENIVQGNDVRFPAGDTHDGWTREMRSKNLLSIAQ 400

CGSWVVITPERQRRDTEGFVDMICKTGAGCGFRLPRPEIVAIQRDGSLEYANKCEDVIARLNPSIIVCVLARKVADRYEA 480

IKKKCCIDRAVPTQVVCARNMTSKSSMSIATKVAIQINCKLGGAPWTVDIPVGGLMVVGYDVCHDTRSKERSFGAMVATL 560

DKAMTKYYSTVNAHTSGEELSSHMSFNIGAALDKYREKNGQLPSRVIIYRDGVGDGQIPYVANHEVVEIKKKLSEIYGGQ 640

PVQLGYIIVSKRINTRLFVNRGRSGDNPKPGTIIDDVVTLPERYDFYLVSQCVRDGTVAPTSYNVIEDTTGLKPDHIQRL 720

TYKLTHLYYNCSNAVRVPSVCQYAHKLAFLAGQSLHGQPHYSLNTSLYFL

Figure S1 Protein sequence of Pxpiwi. The orange and blue color amino acid sequences represent the PAZ and PIWI domain sequence, respectively. The underline amino acid sequences highlight and blue color amino acid glycosylation sites

.

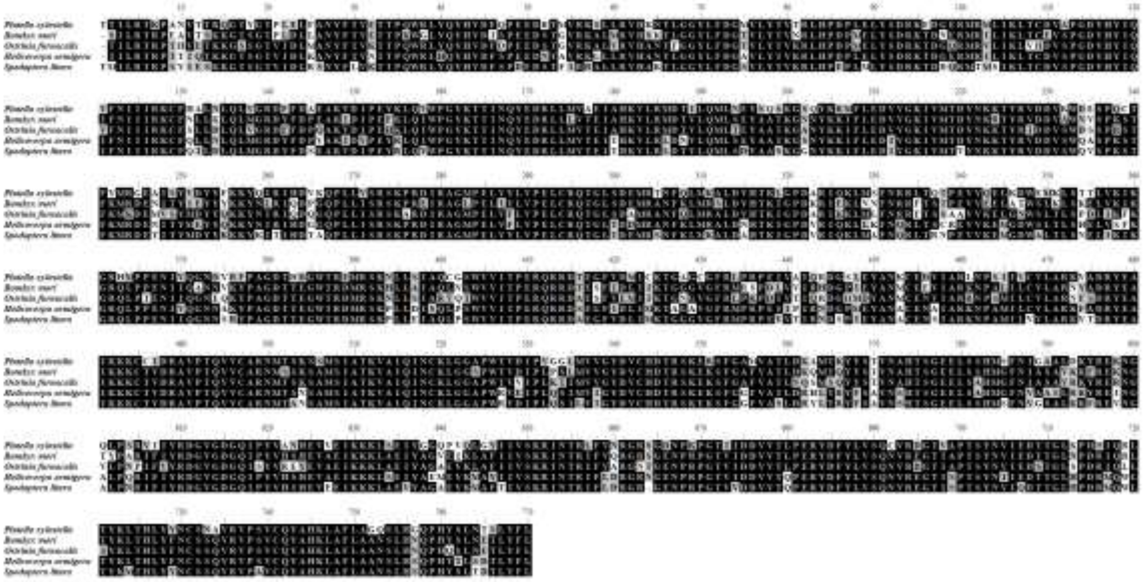

Figure S2 Multiple alignment of piwi amino acid sequences among five lepidopteran species.

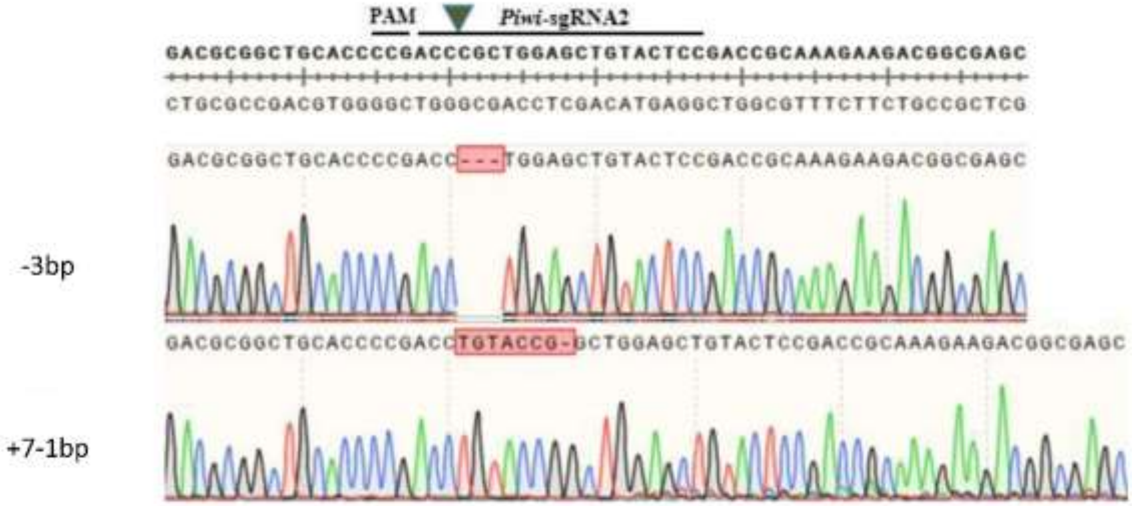

Figure S3 Homozygous mutations of Pxpiwi induced by piwi-sgRNA2. The sgRNA2 target sequences are underlined; the PAM sequences next to sgRNA2 target sequences are also underlined; and the cleavage sites are devoted with a green inverted triangle. Deleted bases are shown as dashes, the inserted bases are shown as red squares.
